# Early life peripheral lipopolysaccharide challenge reprograms catecholaminergic neurons

**DOI:** 10.1038/srep40475

**Published:** 2017-01-10

**Authors:** Lin Kooi Ong, Erin A. Fuller, Luba Sominsky, Deborah M. Hodgson, Peter R. Dunkley, Phillip W. Dickson

**Affiliations:** 1 School of Biomedical Sciences and Pharmacy and Hunter Medical Research Institute, The University of Newcastle, Callaghan, NSW 2308, Australia; 2 Laboratory of Neuroimmunology, School of Psychology and Hunter Medical Research Institute, The University of Newcastle, Callaghan, NSW 2308, Australia; 3 School of Health and Biomedical Sciences, RMIT University, Melbourne, VIC 3083, Australia

## Abstract

Neonatal immune challenge with the bacterial mimetic lipopolysaccharide has the capacity to generate long-term changes in the brain. Neonatal rats were intraperitoneally injected with lipopolysaccharide (0.05 mg/kg) on postnatal day (PND) 3 and again on PND 5. The activation state of tyrosine hydroxylase (TH) was measured in the locus coeruleus, ventral tegmental area and substantia nigra on PND 85. In the locus coeruleus there was an approximately four-fold increase in TH activity. This was accompanied by a significant increase in TH protein together with increased phosphorylation of all three serine residues in the N-terminal region of TH. In the ventral tegmental area, a significant increase in TH activity and increased phosphorylation of the serine 40 residue was seen. Neonatal lipopolysaccharide had no effect on TH activation in the substantia nigra. These results indicate the capacity of a neonatal immune challenge to generate long-term changes in the activation state of TH, in particular in the locus coeruleus. Overall, the current results demonstrate the enduring outcomes of a neonatal immune challenge on specific brain catecholaminergic regions associated with catecholamine synthesis. This highlights a novel mechanism for long-term physiological and behavioural alterations induced by this model.

Early life stress events can exert long lasting programming effects that manifest in adulthood. The bacterial mimetic lipopolysaccharide (LPS) has been used extensively to document the long-lasting effects of a neonatal immune challenge on a variety of physiological and behavioural effects in the adult animal^1^. LPS exposure on postnatal days (PND) 3 and 5 is a well-documented rodent model used to examine the impact of “perinatal programming” on autonomic and hypothalamic-pituitary-adrenal (HPA) stress response systems, the immune system, and the associated long-term behavioural consequences^2^,^3^,^4^,^5^,^6^,^7^. Neonatal inflammatory challenges can activate the sympathoadrenomedullary system leading to release of catecholamines from the adrenal medulla and HPA axis activation resulting in release of glucocorticoids from the adrenal cortex^8^.

Tyrosine hydroxylase (TH) is the rate-limiting enzyme in the biosynthetic pathways for catecholamine synthesis^9^. TH is regulated acutely by phosphorylation at three serine residues (Ser19, Ser31 and Ser40) in the N-terminal regulatory region of TH, and chronically by changes in TH protein synthesis^10^. We previously investigated the effect of neonatal immune challenge at PND 3 and 5 on the sympathoadrenomedullary activation by examining the activation state of TH in the adrenal medulla. Neonatally LPS-treated animals showed significant increases in TH phosphorylation and TH activity up to 24 hours after LPS administration^11^,^12^. Remarkably, this increase in TH phosphorylation and TH activity was maintained into adolescence and adulthood despite there being no further intervention^7^. Such a long-term sustained activation of TH has not been seen in the other stress models that do not involve development. Therefore, this indicates that PND 3 and 5 LPS exposure has a unique capacity to generate long-term changes in the activation state of the catecholamine producing chromaffin cells in the adrenal medulla *in vivo*.

LPS can alter brain catecholamine levels^13^ and the functioning of central immune mediators^1^. It is therefore likely that challenges with inflammatory molecules such as LPS can induce changes in this critical developmental period that are not seen in the adult animals. Given our previous findings regarding peripheral sympathoadrenomedullary and HPA axis hyperactivity, central immunological alterations and long-term behavioural alterations, the current study aimed to investigate the effect of neonatal LPS challenge on the long-term effect on catecholaminergic systems in the brain. We hypothesised that neonatal LPS challenge would induce a long-term activation of TH in the main catecholaminergic nuclei in the brain, the substantia nigra (SN), the ventral tegmental area (VTA) and the locus coeruleus (LC). The current data show that neonatal LPS challenge can produce profound long-term activation of TH in brain catecholaminergic nuclei and that there are major differences in the response of different nuclei suggesting that they are each reprogramed to a different extent by early life LPS challenge.

## Results

### Neonatal peripheral LPS challenge induced long-term alterations in TH, GFAP and Iba-1 protein levels

**Figure 1:**
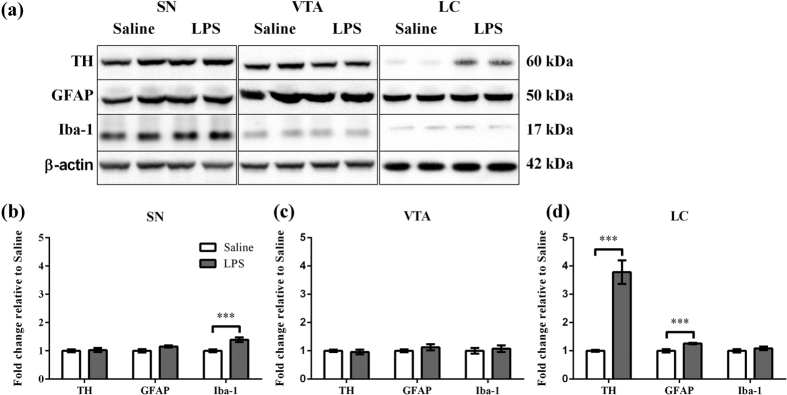
Effect of TH, GFAP and Iba-1 levels in the SN, VTA and LC following neonatal peripheral LPS challenge (Saline, n = 12 and LPS, n = 15). (**a**) Representative immunoblots for TH, GFAP, Iba-1 and β-actin from the SN, VTA and LC comparing the effects of Saline and LPS treatment are shown for two different animals for each treatment. The results of TH, GFAP and Iba-1 levels were calculated relative to β-actin levels from the (**b**) SN, (**c**) VTA and (**d**) LC. Values for Saline and LPS groups were expressed ad fold increase of the mean ± SEM realative to the mean of the Saline group. ***p < 0.001.

The long-term effect of neonatal peripheral LPS challenge in the substantia nigra (SN), ventral tegmental area (VTA) and locus coeruleus (LC) was determined for TH, GFAP (an astrocyte specific cytoskeletal protein marker)^14^ and Iba-1 (microglia calcium homeostasis protein marker)^15^. These parameters were examined at PND 85. TH, GFAP and Iba-1 and each appeared as a single band corresponding to molecular masses of 60, 50 and 17 kDa respectively (Fig. 1a). TH, GFAP and Iba-1 levels were calculated relative to β-actin levels (Fig. 1b–d). In the SN, LPS treatment caused a significant increase in Iba-1 levels (1.4 fold, p < 0.001), but not in TH and GFAP levels relative to Saline treatment (Fig. 1b). In the VTA, there was no effect of LPS treatment on TH, GFAP or Iba-1 levels (Fig. 1c). In the LC, LPS treatment caused a significant increase in TH protein levels (3.8 fold, p < 0.001) and GFAP levels (1.3 fold, p < 0.001), but not in Iba-1 levels relative to Saline treatment (Fig. 1d).

### Neonatal peripheral LPS challenge induced long-term alterations in TH activity and TH phosphorylation

**Figure 2:**
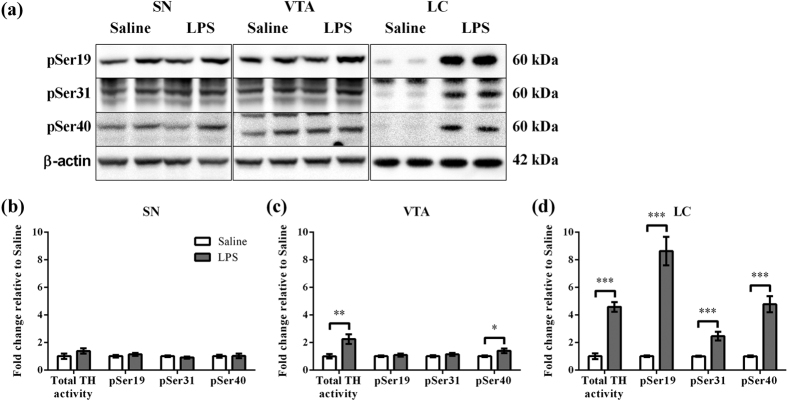
Effect of phospho-TH (pSer19, pSer31 and pSer40) levels in the SN, VTA and LC following neonatal peripheral LPS challenge. (**a**) Representative immunoblots for pSer19, pSer31, pSer40 and β-actin from the SN, VTA and LC comparing the effects of Saline and LPS treatment are shown for two different animals for each treatment. The results of pSer19, pSer31 and pSer40 levels were calculated relative to β-actin levels from the (**b**) SN, (**c**) VTA and (**d**) LC. Values for Saline and LPS groups were expressed as fold increase of the mean ± SEM relative to the mean of the Saline group. *p < 0.05, **p < 0.01, ***p < 0.001.

The effect of neonatal LPS challenge on the TH activation parameters was examined on PND 85 (Fig. 2). As the level of TH protein changed in different brain regions, TH activity levels were calculated as total TH activity by correcting for changes in β-actin levels (Fig. 2b–d). There was a significant increase in total TH activity in the VTA (2.2 fold, p < 0.01) and LC (4.6 fold, p < 0.001), but not in the SN when LPS treatment was compared to Saline. A major mechanism for control of TH activity is the phosphorylation of serine residues in the N-terminal region of TH^10^. We therefore determined the phosphorylation levels of Ser19, Ser31 and Ser40. Again, as the level of TH protein changed in different brain regions, the phosphorylation of the three sites was calculated relative to β-actin levels. Representative immunoblots are shown in Fig. 2a for phospho-TH (pSer19, pSer31 and pSer40) in the SN, VTA and LC after Saline or LPS treatment. In the SN, there was no effect of LPS treatment on phospho-TH levels (Fig. 2b). In the VTA, LPS treatment caused a significant increase in pSer40 levels (1.4 fold, p < 0.05), but not in pSer19 and pSer31 levels relative to Saline treatment (Fig. 2c). In the LC, LPS treatment caused a significant increase in pSer19 (8.6 fold, p < 0.001), pSer31 (2.5 fold, p < 0.001) and pSer40 levels (4.8 fold, p < 0.001) relative to Saline treatment (Fig. 2d).

## Discussion

The major aim of this study was to investigate the long-term consequences of neonatal immune challenge on three different central catecholaminergic nuclei, by examining the activation status of TH and associated glial markers. Our findings indicate that neonatal LPS challenge generates long-term changes in TH activation and glial marker levels in the SN, VTA and LC of adult rats. The changes that we have determined are likely to be due to a direct effect of LPS challenge in inducing a low level inflammation. It is possible though that the sickness of the pups may influence the care given by the mother, however no significant differences existed in weight gain between treatment groups (data not shown), suggesting similar nursing and maternal care between treatment groups. Although we took steps to minimise these issues by having all pups in a litter challenged by LPS or saline (see Animal Protocols), a potential role of differences in maternal care for the LPS challenged pups in generating some of the effects seen cannot be ruled out.

The results show that there was no evidence of long-term changes of TH activation in the SN. This was interesting as the SN was the only brain region that showed a significant sustained increase in Iba-1, a microglial cytoskeletal marker. We have previously reported that neonatal LPS challenge induced increased microglia activation in the hippocampus of adult rats^12^. In a study using a neonatal LPS challenge model but with a much higher LPS dose of 2 mg/kg, Cai *et al*.^16^ demonstrated increases in the microglia activation marker OX42+, a significant decreased expression of TH in the SN, as well as evidence of decreased viability of dopaminergic neurons^16^. Such a high dose of LPS is utilised in adult Parkinson’s disease inflammatory models and results in loss of dopaminergic neurons is the SN^17^. This indicates the potential of LPS to generate long-term responses in the dopaminergic neurons of the SN, but suggests that the LPS dose utilised (0.05 mg/kg) in this study may be too low to produce effects on the TH activation parameters of the SN even though it caused changes in the microglia cytoskeletal marker.

Neonatal LPS challenged animals displayed a significant increase in TH activity in the VTA, which could be due to the significant increase in Ser40 phosphorylation. Ser40 phosphorylation dissociates the bound inhibitory catecholamines and activates TH^9^. Interestingly, these TH alterations were evident without change in the levels of the microglia and astrocyte markers, particularly as microglia and astrocytes are known to be responsive to catecholaminergic stress activation, and the immunoregulatory role of the central dopaminergic system^18^,^19^,^20^. In contrast, there was no change in the level of the TH protein in the VTA. The nature of the changes seen here in the VTA with respect to the mechanism of TH activation is similar to that which we have previously determined in the adrenal gland of adult animals treated with neonatal LPS challenge^7^, that is a chronic increase in TH phosphorylation without any alteration in TH protein levels. With regards to the adrenal gland, there were increases in the phosphorylation of Ser19, Ser31 and Ser40 sites, but the major increase was in the phosphorylation of Ser40. Therefore, the increased activation of TH that we report in the current study could be indicative of an increase in activation of the VTA in response to the neonatal LPS challenge. Consistent with this, we have previously shown that neonatal LPS challenge leads to increased dopamine D2 receptor binding in the nucleus accumbens, a target region of the VTA^21^.

The LC demonstrated significantly pronounced alteration in the TH protein expression, TH phosphorylation of all three sites, as well as TH activation. The increased level of TH protein was accompanied by increased phosphorylation of Ser31 and Ser40, two sites that have been shown to be directly associated with TH activation *in vivo*^7^,^11^,^22^,^23^,^24^,^25^,^26^. This would explain the very significant increase in the LC TH activity. Ser19 phosphorylation is associated with protein binding rather than activation of TH^27^. Ser19 phosphorylation showed the greatest increase in response to neonatal LPS challenge of the three sites, more than double the fold increase in the level of TH protein. We have shown under basal conditions that the stoichiometry of phosphorylation of Ser19 in the LC is 0.35 mol pTH/ mol total TH^23^. This indicates that in the LC of neonatal LPS challenged animals, around 80% of the TH subunits are phosphorylated at Ser19. This has the potential to significantly alter the nature of protein-protein interactions of TH under these conditions. Of all three brain regions studied, increased levels of GFAP were only found in the LC suggesting increased reactive astrocytes in this region^28^, that may have an immunomodulatory effect on catecholaminergic pathway activity, potentially altering behavioural and sympathoadrenomedullary parameters previously demonstrated^7^.

The changes in the LC of neonatal LPS challenged animals can be compared to other *in vivo* stress models. In short-term *in vivo* stress models (less than 1 hour) there were increases in Ser31 and Ser40 phosphorylation in response to social defeat and footshock^23^,^29^ and increases in Ser31 phosphorylation alone in response to restraint, hypotension and glucoprivation^22^,^30^. In contrast to short-term stressor results, the effect of the neonatal LPS challenge on the LC produced much more robust responses in relation to Ser40 phosphorylation and produced a dramatic change in Ser19 phosphorylation that was not seen in the other models. Short-term stress responses are an adaption to an immediate threat but prolonged or repeated stress can be maladaptive. The changes seen in the TH protein levels in the LC in response to the neonatal immune challenge (4 fold) can be put in context by the fact that they are similar to the changes seen in the LC in response to repeated restraint stress over 2 or 6 days (4 to 6 fold)^31^. Therefore, the current findings indicate that the neonatal LPS challenge can produce pronounced long-term changes in the LC that are similar in magnitude to that obtained immediately after what is one of the strongest rodent stress protocols.

The increased activation of the LC can in part explain the activation of the other catecholaminergic cell groups that we have examined. There are both direct and indirect anatomical connections between the LC and the VTA and LC activation can elicit burst firing in the VTA^32^. The LC can activate the pre-ganglionic sympathetic fibres which in turn innervate the adrenal medulla chromaffin cells and activation of the pre-ganglionic sympathetic fibres leads to release of epinephrine and norepinephrine and subsequent requirement for activation of TH^33^. Moreover, the LC sends projections to most brain regions with the exception of the basal ganglia^32^. Therefore the effect of the neonatal LPS challenge in programming the LC to a more activated state has potential to impact on many different brain functions. The findings of the study indicate that neonatal LPS challenge may program central catecholaminergic pathways that are associated with the modulation of endocrine and sympathetic nervous system stress responses. These current outcomes refine and substantiate our previously demonstrated long-term HPA axis, autonomic, and anxiety-like behaviour outcomes using the neonatal LPS challenge model^2^,^7^,^11^,^12^. Importantly, this study suggests a novel mechanism of central catecholaminergic and immunoregulatory pathways mediating the perinatal programming of anxiety-like behaviours and associated pathologies, specifically implicating the catecholaminergic pathways of the LC.

## Materials and Methods

### Antibodies

Total-TH antibody (tTH) and phospho-specific TH antibodies (pSer19, pSer31 and pSer40) were generated and were tested for specificity as described^34^. GFAP antibody (#3670) was purchased from Cell Signaling Technology (Danvers, MA, USA). Iba-1 antibody (AB5076) and anti-goat immunoglobulin (horseradish peroxidase-linked) were purchased from Abcam (Cambridge, UK). β-actin horseradish peroxidase-linked antibody (A3854) were purchased from Sigma-Aldrich. (MO, USA). Anti-sheep antibody (horseradish peroxidise-linked) were purchased from Thermo Fisher Scientific (MA, USA). Anti-rabbit- and anti-mouse-immunoglobulin (horseradish peroxidase-linked) were purchased from Bio-Rad Laboratories (CA, USA). MagicMark™ XP Western Protein Standard was purchased from ThermoFisher Scientific (NSW, Australia).

### Animal Protocols

All animal protocols were approved by the University of Newcastle Animal Care and Ethics Committee and performed in accordance with the New South Wales Animal Research Act and the “Australian code of practice and use of animals for scientific purposes”. Animals were treated as described^7^,^11^. Briefly, Wistar rats were mated at the University of Newcastle. Male neonatal rats were allocated into either saline control (n = 12, derived from 3 litters) or LPS (n = 15, derived from 5 litters) conditions at birth PND 1, with a maximum of 4 pups used per litter. On PND 3 and PND 5, rats were removed from their home cages, weighed and administered intraperitoneally with either 0.05 mg/kg LPS (*Salmonella enterica, serotype Enteritidis*: Sigma-Aldrich, USA in non-pyrogenic saline) or an equal volume of non-pyrogenic 0.9% saline (Livingstone International, Australia). In order to minimise effects of maternal care, pup weights were taken on treatment days, and animals were monitored twice daily for 10 days post-treatment for abnormalities including vocalisation, litter proximity, and nursing. No differences were observed between groups (data not shown). Rats were housed with their dams until PND 22, at which point they were weaned and divided into housing and left undisturbed except for monitoring. Rats were euthanized on PND 85 with a lethal injection of sodium pentobarbital (200 mg/kg, Virbac, Pty. Ltd, Milperra, Australia).

The SN, VTA and LC were dissected from the coronal sections as previously described which will separate out the different brain sections such that they only have the ascribed catecholaminergic nuclei^22^,^23^. The SN, VTA and LC samples were separately processed as previously described^23^. Brain samples were sonicated in homogenizing buffer (2 mM potassium phosphate buffer, 1 mM EGTA, 1 mM EDTA, 1 protease inhibitor cocktail tablet, 1 PhosStop tablet, 1 mM DTT, 80 μM ammonium molybdate, 1 mM sodium pyrophosphate, 1 mM sodium vanadate, 5 mM β-glycerolphosphate, 2 μM microcystin, pH 7.4) with a microsonicator (UP50H, Hielscher Ultrasonics GmbH, Teltow, Germany). Samples were centrifuged at 14 000 g for 20 min at 4 °C. The clear supernatants were collected and protein concentrations were determined. Samples were aliquoted into two equal volumes. One aliquot of each sample was mixed with sample glycerol buffer (2% sodium dodecyl sulfate, 50 mM Tris, 10% glycerol, 1% DTT, 0.1% bromophenol blue, pH 6.8) and this was used for western blot. The second aliquot from the same sample was used for tritiated water release assay.

### Western blot

Western blot was performed as previously described with some modifications^23^. Samples (30 μg of total tissue protein), protein standard and TH specific positive controls were subjected to NuPAGE Novex 4–12% Bis-Tris Midi Gels. Gels were transferred to nitrocellulose membranes by western blotting in boric acid transfer buffer (50 mM boric acid, 2 mM EDTA, pH 8.9). Nitrocellulose membranes were washed in Tris-buffered saline with tween (TBST) (150 mM NaCl, 10 mM Tris, 0.075% Tween-20, pH 7.5) and blocked in 5% skim milk powder (SMP) in TBST for 1 h at 25 °C. Membranes were incubated with primary antibodies (tTH; 1:5000 in 1% SMP, GFAP; 1:5000 in 5% SMP, Iba-1; 1:1000, pSer19; 1:2000 in 1% SMP, pSer31; 1:500 in 1% SMP, pSer40; 1:1000 in 1% SMP for overnight at 4 °C or β-actin horseradish peroxidase-linked antibody; 1:50,000 for 1 h at 25 °C) and horseradish peroxidase-linked anti-IgG secondary specific antibodies (anti-rabbit; 1:7500, anti-mouse; 1:10000, anti-sheep; 1:7500, anti-goat; 1:5000) for 1 h at 25 °C. In between each incubation step, membranes were washed in TBST. Membranes were visualized on Fugifilm Las-3000 imaging system (Fuji Film, CT, USA) using Luminata Classico detection reagents. The density of the bands was measured using MultiGauge V3.0 (Fuji Film). TH, GFAP, Iba-1 (Fig. 1b–d) and phospho-TH (pSer19, pSer31 and pSer40) (Fig. 2b–d) levels were normalized to β-actin levels and expressed as fold change relative to the Saline samples. Full-length blots were presented in Sup Fig. 1 (for SN), Sup Fig. 2 (for VTA) and Sup Fig. 3 (for LC).

### Tritiated water release assay

Tritiated water release assay was performed as previously described (Ong *et al*.^23^). Samples (50 μg of total tissue protein) were mixed in reaction mixture (2 mM potassium phosphate, 36 μg catalase, 0.008% β-mercaptoethanol, 24 μM L-tyrosine, 1 μCi 3, 5-[3 H]-L-tyrosine, pH 7.4). The reactions were initiated with the addition of 100 μM tetrahydrobiopterin in 5 mM HCl. Control representing background reactions were added with 5 mM HCl but did not contain tetrahydrobiopterin. Assays were performed for 20 min at 30 °C and were stopped by addition of 700 μL charcoal slurry (7.5% activated charcoal in 1 M HCl). Mixtures were vortexed for 1 min and were centrifuged at 14 000 g for 10 min at 30 °C. 350 μL supernatants were added to 3 mL scintillation cocktail and were vortexed for 10 s. Mixtures were assayed by Liquid Scintillation Analyzer (Tri-Carb 2810 TR, PerkinElmer) for 10 min per sample. The assessment of TH protein, phospho-TH and TH activity was conducted on the same sample. Therefore, the changes in total TH activity levels (Fig. 2b–d) were normalized to β-actin levels and expressed as fold change relative to the Saline samples.

### Statistical analysis

The data for Saline and LPS groups were expressed as a fold change of the mean ± SEM to the mean of the Saline group. These data were analysed by using Prism 6 for Windows (Version 6.01, GraphPad Software, Inc., La Jolla, CA, USA). The data were analysed using two-tailed unpaired parametric Student’s *t*-test. All differences were considered to be significant at p < 0.05.

## Additional Information

**How to cite this article**: Ong, L. K. *et al*. Early life peripheral lipopolysaccharide challenge reprograms catecholaminergic neurons. *Sci. Rep.* **7**, 40475; doi: 10.1038/srep40475 (2017).

**Publisher's note:** Springer Nature remains neutral with regard to jurisdictional claims in published maps and institutional affiliations.

## Supplementary Material

Supplementary Information
