# Supplementary material for: Early life peripheral lipopolysaccharide challenge reprograms catecholaminergic neurons
Source: Sci Rep. 2017 Jan 10;7:40475. doi: 10.1038/srep40475 (PMC5223129; doi:10.1038/srep40475)
Supplement: Supplementary Information [file srep40475-s1.pdf]

Supplementary information

Manuscript title

Early life peripheral lipopolysaccharide challenge reprograms catecholaminergic neurons

Author list

Lin Kooi Ong, Erin A. Fuller, Luba Sominsky, Deborah M. Hodgson Peter R. Dunkley, Phillip W. Dickson

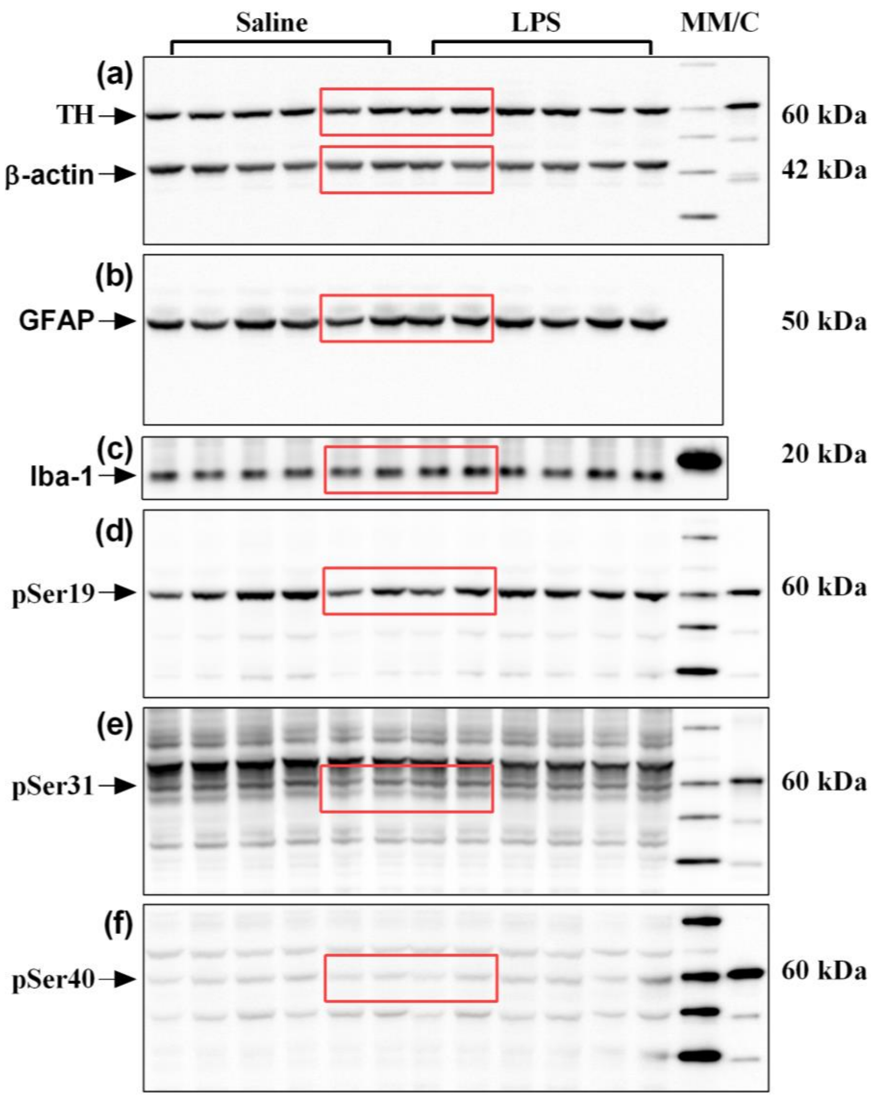

Sup Fig. 1: Full-length SN immunoblots probed with total-/phospho-TH, GFAP, Iba-1 or β-actin antibodies. MM, MagicMark™ XP Western Protein Standard; C, recombinant TH protein (specifically phosphorylated at Ser19, Ser31 or Ser40); red box, cropped images represented in Fig. 1 and Fig. 2.

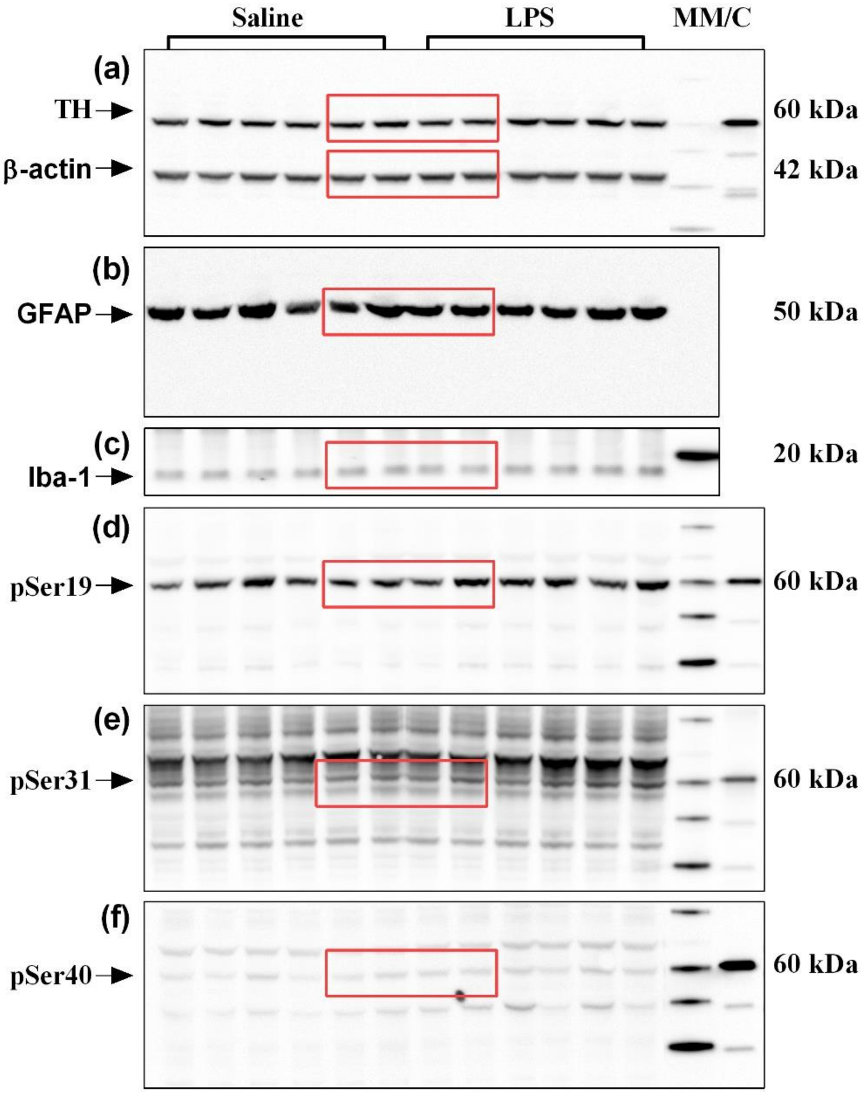

Sup Fig. 2: Full-length VTA immunoblots probed with total-/phospho-TH, GFAP, Iba-1 or β-actin antibodies. MM, MagicMark™ XP Western Protein Standard; C, recombinant TH protein (specifically phosphorylated at Ser19, Ser31 or Ser40); red box, cropped images represented in Fig. 1 and Fig. 2.

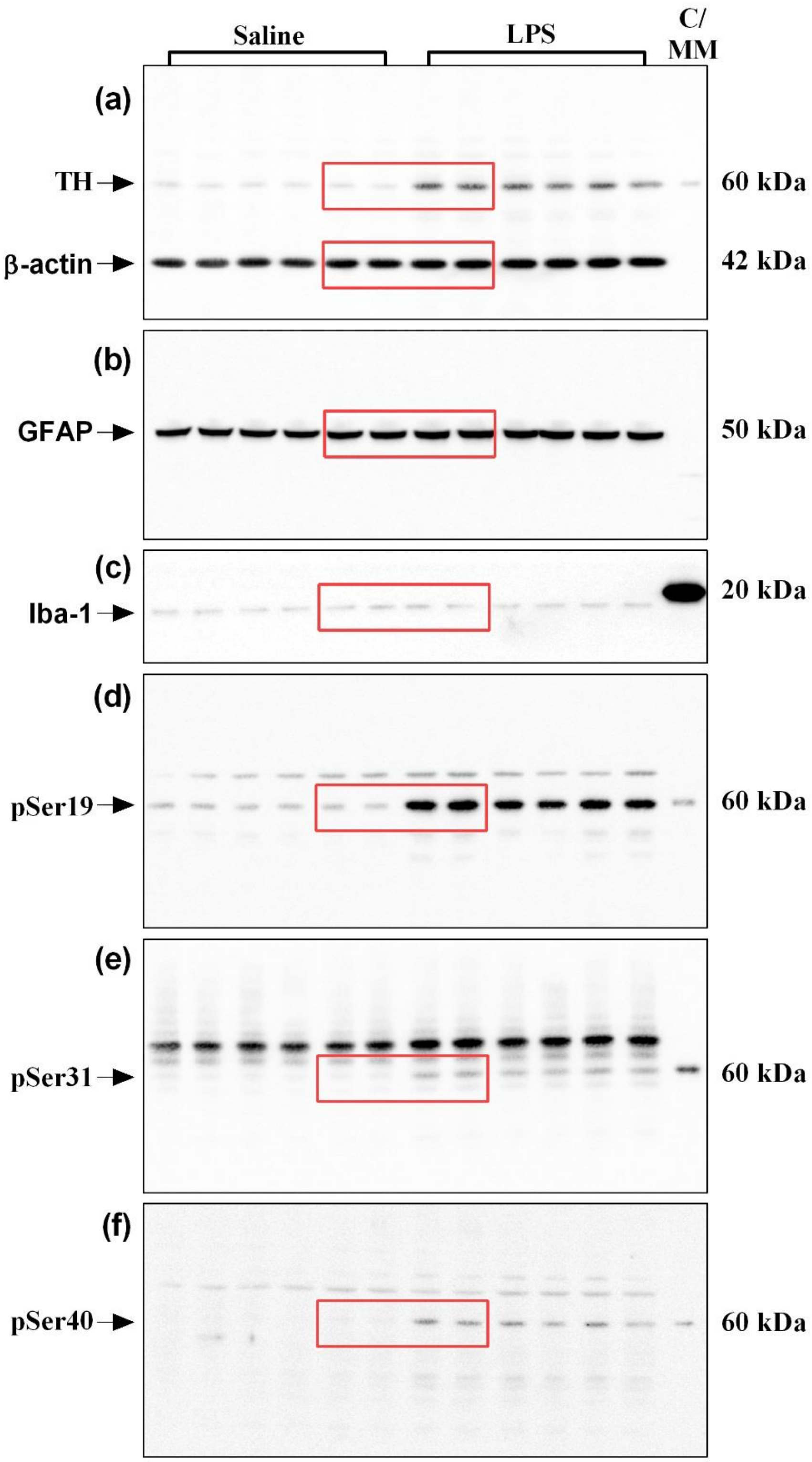

Sup Fig. 3: Full-length LC immunoblots probed with total-/phospho-TH, GFAP, Iba-1 or β-actin antibodies. MM, MagicMark™ XP Western Protein Standard; C, recombinant TH protein (specifically phosphorylated at Ser19, Ser31 or Ser40); red box, cropped images represented in Fig. 1 and Fig. 2.
